# The high-field magnet endstation for X-ray magnetic dichroism experiments at ESRF soft X-ray beamline ID32

**DOI:** 10.1107/S160057751600179X

**Published:** 2016-02-20

**Authors:** K. Kummer, A. Fondacaro, E. Jimenez, E. Velez-Fort, A. Amorese, M. Aspbury, F. Yakhou-Harris, P. van der Linden, N. B. Brookes

**Affiliations:** a European Synchrotron Radiation Facility, 71 Avenue des Martyrs, CS40220, F-38043 Grenoble Cedex 9, France

**Keywords:** high-field magnet, endstation, ID32, ESRF, sample preparation, XMCD, XMLD

## Abstract

A new high-field magnet endstation for X-ray magnetic dichroism experiments has been installed and commissioned at the ESRF soft X-ray beamline ID32. The magnet consists of two split-pairs of superconducting coils which can generate up to 9 T along the beam and up to 4 T orthogonal to the beam. It is connected to a cluster of ultra-high-vacuum chambers that offer a comprehensive set of surface preparation and characterization techniques. The endstation and the beam properties have been designed to provide optimum experimental conditions for X-ray magnetic linear and circular dichroism experiments in the soft X-ray range between 400 and 1600 eV photon energy. User operation started in November 2014.

## Abstract 2

The high-field magnet endstation for X-ray magnetic dichroism experiments at the ESRF soft X-ray beamline ID32 is presented.

## 1. Introduction   

The former ESRF soft X-ray beamline ID08 had a long-standing user program in X-ray absorption in high magnetic fields. The experimental setup was one of the first where a high-field magnet synchrotron endstation for X-ray magnetic dichroism measurements was combined with a sample preparation system that allowed one to grow and characterize samples *in situ*. This combination turned out to be extremely useful for many experiments (Dhesi *et al.*, 2001 ▸; Gambardella *et al.*, 2002*a* ▸,*b* ▸, 2003 ▸, 2009 ▸; Ohresser *et al.*, 2005 ▸; Umbach *et al.*, 2012 ▸; Honolka *et al.*, 2012 ▸; Eelbo *et al.*, 2013 ▸; Stepanow *et al.*, 2014 ▸; Vijayaraghavan *et al.*, 2015 ▸). Today, many soft X-ray XAS beamlines are equipped with similar setups, *e.g.* the beamlines BOREAS (ALBA), DEIMOS (Soleil), X-Treme (PSI-SLS; Piamonteze *et al.*, 2012 ▸). A major disadvantage of the ID08 system was the monolithic design of the sample preparation system concentrating all functionalities in one vacuum chamber. This made interventions for repairs and upgrades difficult and the system rather inflexible. It was also only possible to apply a magnetic field of up to *B* = ±5 T in one direction only, either parallel or perpendicular to the beam. In order to change the field direction the ultra-high vacuum (UHV) magnet had to be disconnected from the beamline as well as the sample preparation chamber and was then rotated by 90° and reconnected.

With the ESRF Upgrade Program Phase I the former beamline ID08 has been replaced by a new soft X-ray beamline, ID32 (http://www.esrf.fr/UsersAndScience/Experiments/EMD/ID32/). On one of the two branches of this beamline the X-ray absorption spectroscopy (XAS) user program of ID08 is being continued. In the process of the upgrade, a new high-field magnet endstation has been installed and connected in UHV to a cluster of UHV chambers which offer a wide arsenal of sample preparation techniques. The beam properties and stability of the new beamline surpass the figures achieved at ID08 and provide better conditions for X-ray magnetic circular dichroism (XMCD) and X-ray magnetic linear dichroism (XMLD) experiments in the soft X-ray range. Here we give a description of the endstation and the sample preparation system and show some typical experimental data obtained with the new setup.

## 2. Description of the endstation   

**Figure 1:**
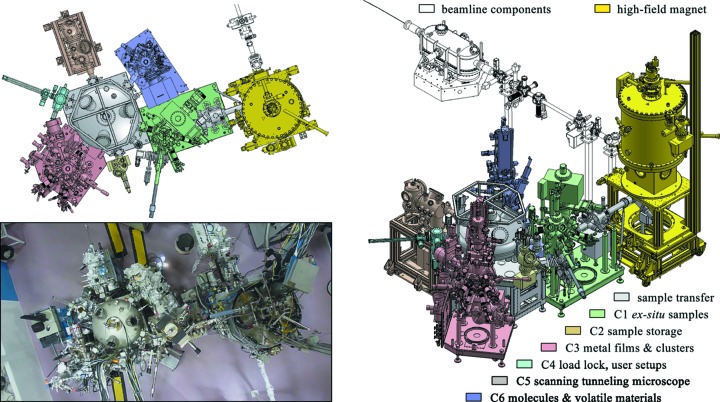
Schematic representation and photograph of the high-field magnet endstation and the UHV sample preparation system.

The new high-field magnet endstation is designed for XMCD and XMLD experiments on a wide variety of samples. It consists of the high-field magnet itself and a cluster of six UHV chambers for *in situ* sample preparation and characterization. Samples can be transferred between the magnet and the sample preparation facilities in UHV. The entire setup is placed in a designated experimental cabin and takes up about 3 m × 5 m of floor space. A schematic representation of the endstation and the sample preparation facilities is shown in Fig. 1 ▸.

### 2.1. Beam properties and X-ray absorption scans   

The new beamline, ID32, delivers polarized X-rays in the energy range from 400 eV to 1600 eV, covering the transition metal *L*-edges and the rare-earth *M*-edges. Up to three APPLE-II type undulators can be used at the same time, always working at their first harmonic and providing close to 100% linear and circular polarized light. Refocusing optics using mechanical benders allow independent and continuous variation of the beam size at the sample in the horizontal and vertical from 50 µm × 10 µm up to about 2.5 mm × 1.5 mm. The beam size can be redefined with a set of baffle slits after the refocusing optics if needed. In combination the mechanically bent optics and baffle slits make it possible to reach a wide range of beam sizes and photon densities on the sample which makes the beamline suitable for a wide range of materials, from radiation-sensitive samples like molecular magnets which require large spots and low photon densities to micrometer-sized photon-hungry samples.

The resolving power 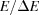 of the beamline over the entire energy range exceeds 5000 at the nominal exit slit size of 50 µm. The energy drifts observed over one week are less than 100 meV, typically smaller, and can be corrected using a reference signal (see §2.4).

As on the previous ID08 beamline, X-ray absorption scans can be taken on-the-fly, *i.e.* the monochromator and the undulator gap are scanned synchronously and data are taken continuously. This dramatically reduces acquisition times compared with point-by-point scans (Rogalev *et al.*, 1998 ▸; Joly *et al.*, 2014 ▸). A scan across 80 eV with a step size of 100 meV and a counting time of 100 ms per point, which are typical parameters during user experiments, takes less than 85 s execution time in total, 80 s of which are data acquisition.

A more detailed description of the optical layout of the beamline and the beam properties at the sample will be given elsewhere (Brookes *et al.*, 2016 ▸).

### 2.2. High-field superconducting magnet   

The UHV high-field magnet was built by Cryogenic Limited (http://cryogenic.co.uk). It consists of two sets of split-pair superconducting coils which can generate a magnetic field of up to 9 T along and up to 4 T orthogonal to the beam, in the horizontal direction (Cezar *et al.*, 2013 ▸). The 9 T split-pair consists of four coaxial coils: an inner Nb_3_Sn coil and an outer NbTi coil on either side of the split. The four coils are connected in series. The 4 T split-pair is made of one NbTi coil on either side of the split, also operated in series. The coils are embedded in a liquid-He (LHe) bath keeping them at 4.2 K, well below the critical temperature for superconductivity. The magnet has been delivered with an integrated protection circuit that should fully protect it in the event of a quench but with no interlock to the LHe level. Such an interlock is currently being implemented.

Operation of the 9 T and 4 T coils is exclusive OR, *i.e.* only one set of coils can be used at a time. The magnetic field can be swept at high rates of up to 8 T min^−1^ for the 9 T coils and up to 2 T min^−1^ for the 4 T coils although the coils are typically operated at 3 T min^−1^ and 1 T min^−1^, respectively, in order to reduce the risk of quenches. Fast sweep rates are important for synchrotron applications where a minimum of dead-time is desired to use the available beam time efficiently.

**Figure 2:**
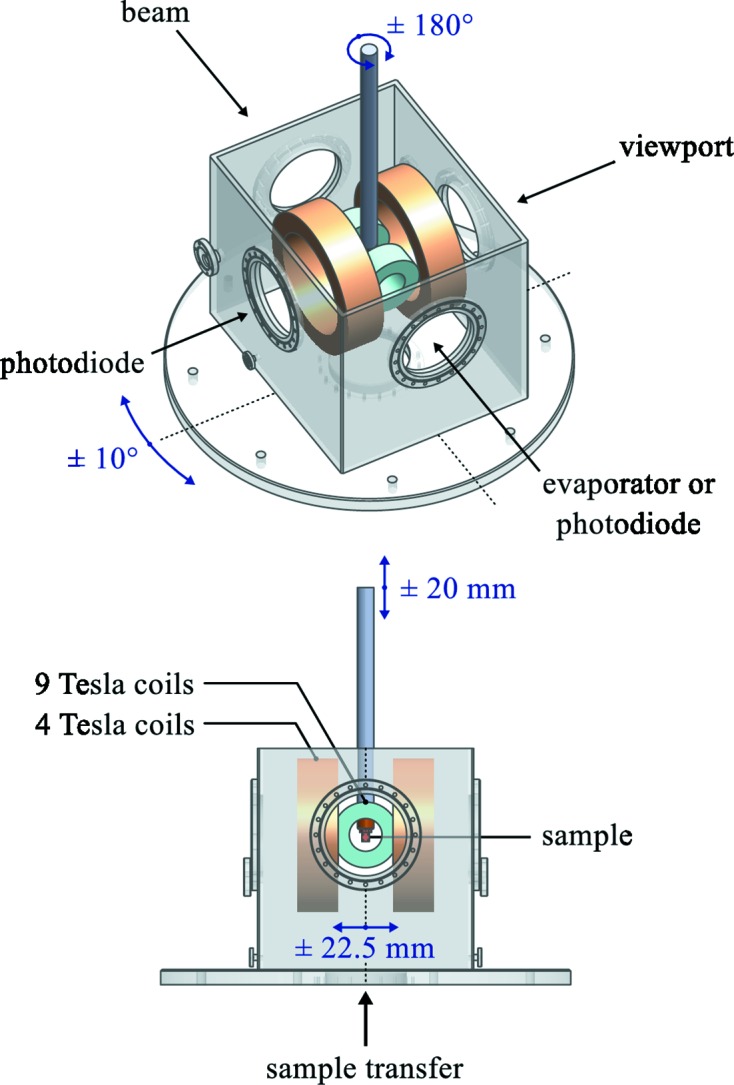
Schematic representation of the high-field magnet UHV chamber. The sample is enclosed by thermal shields. All ports have provisions to reduce the thermal radiation load (not shown).

A schematic representation of the high-field magnet is shown in Fig. 2 ▸. The variable temperature insert is mounted vertically and allows ±180° rotation of the sample about the vertical axis and ±20 mm sample translation along the vertical axis. In the horizontal the sample is translated together with the entire magnet which is mounted on a translation stage. In order to improve the electron yield signal with an orthogonal field applied the entire magnet can be rotated by ±10°. A photodiode is mounted inside the magnet at 90° with respect to the beam axis. At the back of the magnet a DN40CF port is available for mounting an evaporator and evaporating at low temperatures onto the sample. It can also be used to mount a second photodiode for measurements in transmission mode. The sample transfer into the magnet is done from the bottom. Top-post cleaving at low temperature inside the magnet is possible. The sample is protected from thermal radiation by silver-plated thermal shields cooled to 100 K by the liquid-nitrogen (LN2) reservoir. The ports at the bottom and the back of the magnet can be closed with movable shields and the view port is shielded with a sapphire screen, all kept at 100 K. The beam port is equipped with a movable aperture at 100 K that allows one to reduce the open area down to a few mm^2^. The photodiode is thermally connected to the LN2 shields and thus kept at cryogenic temperatures. The thermal shielding has a big effect on the base sample temperature which is 5 K with the shields closed compared with 8 K with the shields open.

### 2.3. Variable temperature insert (VTI)   

The magnet is equipped with a He^4^ continuous-flow cryostat. The VTI has been delivered by Cryogenics together with the magnet. It is connected by an external siphon to the main LHe reservoir which facilitates interventions on the VTI considerably. The VTI can be operated at cryogenic temperatures down to 1.4 K and up to 400 K on the heat exchanger. The lowest possible temperature on the sample to date is 5 K. Preliminary tests show that this can be reduced in the future to about 3 K by adding a thermal shield to the bottom of the VTI. Cooling down to and warming up from base temperature takes less than 30 min. Typically cooling and heating cycles are shown in the report of the factory acceptance tests by Cezar *et al.* (2013 ▸). In day-to-day user operation the magnet is refilled once a day with LHe and LN2. If needed, the 100 l LHe reservoir allows the magnet to be used for two consecutive days under typical experimental conditions before running out. The base pressure in the magnet during experiments is better than 2 × 10^−10^ mbar, typically in the 10^−11^ mbar range.

**Figure 3:**
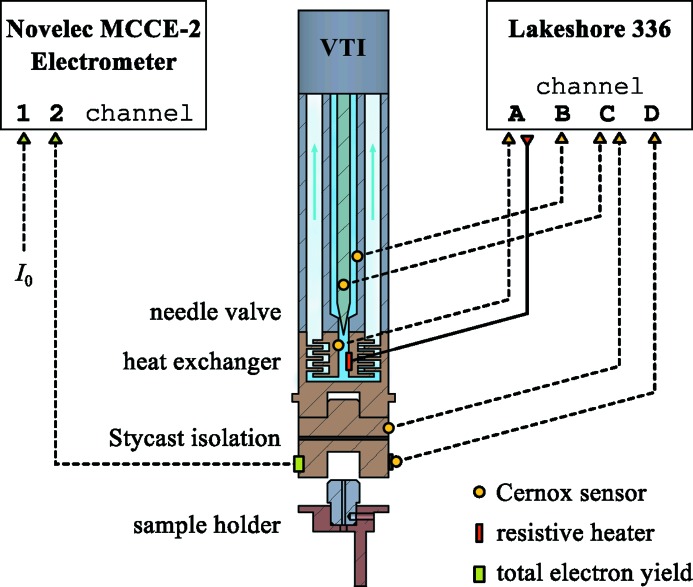
Schematic of the tail of the VTI showing the position of the temperature sensors and resistive heaters as well as the electrical insulation and contact for the sample drain current measurements.

A scheme of the tail of the VTI is shown in Fig. 3 ▸. LHe is transferred from the LHe tank of the magnet *via* the external siphon into the VTI. A reservoir of LHe before the needle valve is always kept filled with LHe in order to reduce cooling times when going from room to low temperatures. The needle valve is motorized, equipped with an encoder and controlled remotely. After the needle valve the LHe passes the heat exchanger where it evaporates and is pumped out through the exhaust. The temperatures of the helium reservoir, the needle valve and the heat exchanger are monitored using Cernox temperature sensors and a Lakeshore 336 temperature controller. A resistive heater on the heat exchanger allows temperature control with a PID control loop on the heat-exchanger temperature sensor. The heater and the temperature sensors are not in direct contact with the Cu tail of the VTI but separated by LHe or He gas. This prevents the introduction of electrical noise on the sample current signal when operated. We do not observe any change in the signal-to-noise ratio of the sample current signal when the temperature sensors and heaters are operated or not (see §3.3).

At the end of the tail of the VTI a home-made Cu–Stycast–Cu interface for electrical insulation of the sample is mounted. Previously, we had used a sapphire disk instead of Stycast for electrical insulation but found that the sapphire develops horizontal cracks upon thermal cycling which considerably reduces its thermal conductivity. The matched thermal expansion coefficients of Cu and Stycast seem to avoid this problem. However, the low glass transition temperature of Stycast of around 350 K, at which the electrical resistivity dramatically drops and the interface becomes poorly conducting, makes it currently impossible to reach the targeted sample temperature of 400 K.

The sample is fixed with a screw-in mechanism at the end of the Cu–Stycast–Cu interface. In order to read the sample temperature and the temperature difference across the Stycast layer, the interface is equipped with two Cernox temperature sensors on either side of the Stycast. Even though good care of proper electrical insulation has been taken when installing the two sensors, we find that both can introduce noise on the sample signal. During an absorption scan these two sensors are switched off remotely and reactivated afterwards.

Starting from base temperature, any temperature up to the maximum available sample temperature of 320 K can be reached at any ramp rate and be stabilized using just the heater on the heat exchanger. No adjustments to the needle valve settings are required. Going from high to low temperature requires a more open needle valve, with the opening depending on the desired cooling rate. Ramp rates and temperature stabilization can again be controlled with the heater on the heat exchanger. The typical time to stabilize a sample temperature is of the order of 20 min.

### 2.4. Detection scheme   

Both the total electron yield (TEY) and the total fluorescence yield (TFY) signals are measured during X-ray absorption scans. For monitoring and normalization to the incident photon flux we also record the current on the last mirror and a signal from a dedicated *I*_0_ monitor which uses either a gold mesh or a thin diamond film depending on the experiment (Kummer *et al.*, 2013 ▸). Furthermore, a signal from oxide reference samples is taken with each absorption scan. This signal is not affected by changes in polarization, temperature or magnetic field and can be used to identify and correct possible energy drifts of the beamline. The detection scheme is designed to provide extremely low noise signals under all experimental conditions and at any temperature. XMCD signals of less than a few tenths of a percent have been measured repeatedly with good quality in several user experiments. On-the-fly energy scans and on-the-fly magnetic field scans for magnetization measurements allow very fast data collection (see §3.1 and §3.2 below).

The TEY measurement is realised by measuring the drain current from the sample. On the sample side the electrical contact is at the end of the VTI tail, directly above the sample (Fig. 3 ▸). It is connected to a UHV connector at the top of the VTI with a shielded Cu wire going through a dedicated tube in the VTI that acts as a Faraday cage. All other wires for temperature sensors are going through another tube at the opposite side of the VTI. Outside vacuum the signal is fed through a triax cable into a Novelec EPV HS electrometer positioned above the magnet and controlled by an Novelec MCCE2 module. The *I*_0_ signal is measured with an identical Novelec electrometer connected to the second channel of the MCCE2 module. We found that having both electrometers on the same MCCE2 module significantly improves the signal-to-noise in the sample current divided by the *I*_0_ signal.

The TFY is measured with an IRD photodiode (AXUV57C-EUT) mounted at 90° with respect to the incident beam. The diode has a ceramic casing to reduce the amount of ferromagnetic materials used. The sensor is eutectically mounted so that it is UHV-compatible up to the 10^−10^ mbar range. It can also be baked to 120°C. The chip itself has a size of 24 mm × 24 mm. The total lateral size of the package is 31 mm × 31 mm in size (44 mm on the diagonal) which is small enough for the re-entrant tubes in the magnet. The full package is thermally connected to the LN2-cooled shrouds in order to reduce thermal radiation onto the sample. The diode is protected by a double Al filter window against visible light, ions and electrons from the sample. The Al foils are 0.4 µm thick and doubled to reduce the pinholes. This results in about 20% transmission at 500 eV and 78% at 1000 eV. The signal of the photodiode is transferred by two Kapton-coated wires to a DB9 UHV connector from which it is fed either into a Keithley 427 or a Novelec current amplifier using a triax cable.

All signals are transmitted *via* fiber optic from the current amplifier to an ESRF P201 counting card in the data acquisition PC. They are always measured on a constant offset signal which is frequently measured and subtracted from the signals in software. Using an offset avoids artifacts in the voltage-to-frequency conversion when the signal is oscillating around zero.

Repeated tests of different current amplifiers under experimental conditions at the new ID32 endstation and its ID08 predecessor have shown that the Novelec electrometers achieve an excellent signal-to-noise ratio which we did not reach or surpass with other low-noise current amplifiers, like the Keithley 428 or the FEMTO DLPCA-200. Unfortunately, the production of the Novelec as well as the Keithley 428 current amplifiers has been discontinued. In our opinion, there is currently a real need for very low noise current amplifiers with variable gain which has also been identified at other synchrotrons and led to in-house developments (Lidon-Simon *et al.*, 2012 ▸).

### 2.5. Sample preparation facilities   

The sample preparation system consists of six UHV chambers arranged around a circular distribution chamber (Fig. 1 ▸). It is connected in UHV with the high-field magnet *via* a 1.5 m transfer tunnel. The transfer arm of the circular distribution chamber is automatized. All other transfers are realised with wobble sticks, magnetic transfer rods and, for the transfer tunnel, a UHV vacuum train. The transfer system and two of the UHV chambers were built by Prevac (https://www.prevac.eu/). The other four chambers were designed in-house. The base pressure in the entire system is better than 2 × 10^−9^ mbar with most of the chambers being in the low 10^−10^ mbar pressure range. Each of the six chambers, C1 to C6, offers a different functionality. The large distance between the sample preparation chambers and the UHV magnet allows sample preparation without being affected by stray fields when the magnet is operated.

Three Omicron EFM single evaporators, one Omicron EFM triple evaporator (Scienta Omicron GmbH, http://www.scientaomicron.com/en/home) and one Dodecon 4x OMBE molecule evaporator for up to four evaporants (Dodecon Nanotechnology GmbH, http://www.dodecon.de) are available to the users for their experiments. A vacuum glove box will become available in the near future. The system is flexible enough for future changes and further additions if needed. Below we give a brief description of each of the six UHV chambers and their purpose.

*C1 *ex situ* samples.* C1 is directly connected to the endstation *via* a 1.5 m transfer tunnel and allows loading, storing and basic preparation of *ex situ* samples. It is equipped with a load lock that allows loading two sample holders at a time. For basic sample treatment a sputter gun and a heater going up to 450°C are available. Scrapers, cleavers and evaporators can be mounted either on this chamber or in the chamber directly below the magnet depending on the users’ needs. Up to five samples can be stored on a built-in storage. This chamber is also used to transfer the Omicron-type sample plates used in C2 to C6 onto the sample holders for the high-field magnet endstation (see §2.6).

*C2 sample storage.* Storage space for up to 20 Omicron-type sample plates.

*C3 metal films and clusters.* C3 is configured for the growth and characterization of metal and oxide films and clusters. It has an ARPES-type configuration with two UHV chambers stacked vertically and a UHV manipulator mounted on top. The upper chamber is for sample preparation. It offers an ion sputter gun, four ports for UHV evaporators which can be retracted and changed without breaking vacuum, and an electron-beam heater reaching up to 2000°C. A resistive heater reaching up to 1200°C and a quartz crystal microbalance (QCM) are mounted directly on the manipulator. A motorized mask on the manipulator allows evaporating wedges onto the sample. The manipulator can be cooled with LN2 to below 100 K. A parking stage in the upper chamber allows parking of up to two samples at room or LN2 temperatures. The lower chamber is equipped with a conventional VG LEED and a STAIB DESA 100 double-pass CMA (STAIB Instruments GmbH, http://www.staibinstruments.com/) for Auger electron spectroscopy (AES) allowing the grown samples to be characterized.

*C4 load lock, user setups.* C4 is a small module with a two-stage setup. The first chamber is a small load lock allowing two Omicron-type sample plates to be loaded at a time. It is connected with a DN40CF valve to a second chamber which interfaces with the transfer system of the circular distribution chamber. The load lock can be taken off and replaced by user setups or UHV suitcases as long as Omicron-type sample plates are used.

*C5 scanning tunneling microscope (STM).* C5 is built around an Omicron VT-STM which can be operated at room temperature and at cryogenic temperatures down to about 70 K. A noise level of <2 Å peak-to-valley is routinely achieved with all pumps of the adjacent chambers running. The chamber also contains an Omicron STM tip conditioning tool which allows oxidized STM tips to be reconditioned.

*C6 molecules.* C6 has the same two chamber configuration as C3 but is dedicated to deposition of molecules and other volatile materials which tend to contaminate UHV chambers. It is equipped with the same type of manipulator as C3 allowing one to cool the sample to below 100 K and heat it up to 1200°C. The manipulator is also equipped with the QCM and the motorized mask for wedge growth. The upper chamber offers four ports facing up for retractable evaporators and an ion sputter gun for cleaning surfaces. The lower chamber is equipped with a conventional Omicron SPECTALEED and an Omicron MCP-LEED working at a few µA sample current for systems which are very sensitive to electron-beam damage such as molecular magnets.

### 2.6. Sample holders   

**Figure 4:**
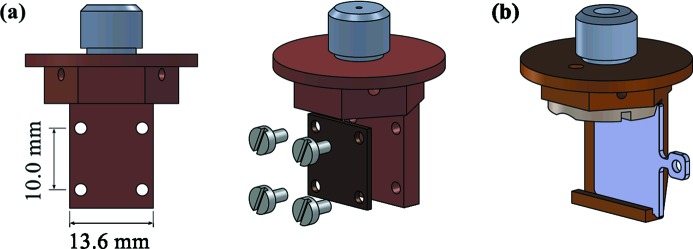
(*a*) Standard sample holder for *ex situ* samples. (*b*) Sample holder for Omicron-type sample plates used in the sample preparation system.

Two types of sample holders are used in the high-field magnet (Fig. 4 ▸). In both cases the transfer relies on an M10 thread screw-in system with the male part on the shuttle and an M10 threaded hole in the bottom of the VTI. The standard sample holder offers a 14 mm × 14 mm area for mounting samples. Samples can be pre-mounted in the users’ home institution on simple sample plates which screw on the sample holders. A special variant of this sample holder allows mounting and tight clamping of the Omicron-type sample plates used in the sample preparation system. The transfer and clamping of the sample plates on the shuttles is carried out in the C1 chamber using a wobble stick and an in-vacuum Allen key tool.

## 3. Scientific applications   

The high-field magnet endstation is primarily designed for X-ray magnetic dichroism experiments aiming at element-specific characterization of the magnetic properties at surfaces, interfaces and in bulk systems. The research of the user community includes molecular magnets, topological insulators, magnetic impurities, exchange bias systems and single-crystalline systems. Here we show a few examples of typical experimental data to demonstrate the capabilities of the endstation and the sample preparation facilities. We do not discuss the scientific outcome in detail but rather focus on the experimental details. The system has been set up for fast data acquisition of very high quality under all experimental conditions. In the first few user experiments on the high-field magnet endstation XMCD signals of a few tenths of a percent of the total absorption were measured with high quality (Zhiwei, 2015 ▸). To achieve this goal we compromised on the lowest possible sample temperature which is currently limited to 5 K with the ultimate target of 3 K.

### 3.1. Step edge decoration of the Cu(111) surface with cobalt: sample preparation and magnetic characterization   

A typical surface science experiment at a synchrotron often requires repeating the sample preparation protocols established in the laboratory. At the ID32 endstation up to one week for sample preparation can be given to the users before the start of their official beam time. This ensures that the users have gained good control over their sample preparation before starting their beam time.

**Figure 5:**
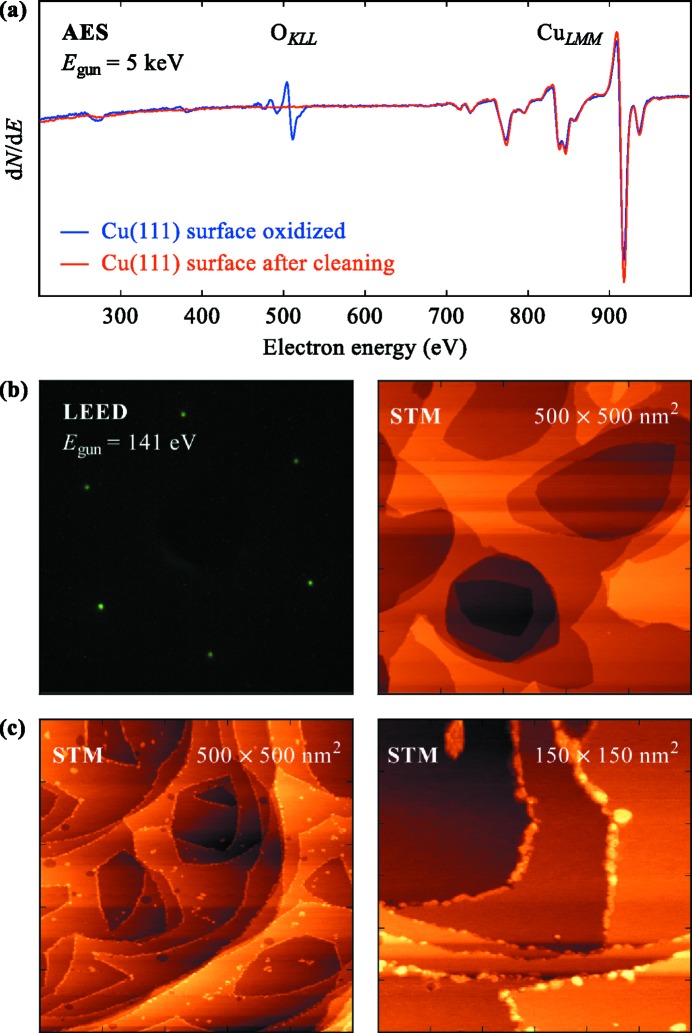
Preparation of a Cu(111) surface with Co decorated step edges. (*a*) AES before and after cleaning the Cu(111) surface. (*b*) LEED and STM show the cleanliness of the Cu surface after several cycles of sputtering and annealing. (*c*) STM images of the Cu surface after Co deposition.

Fig. 5 ▸ shows a typical example of preparing a surface science sample. In this experiment the previously reported decoration of Cu(111) step edges with iron or cobalt clusters (Speller *et al.*, 1998 ▸; Repain *et al.*, 2000 ▸; Chang *et al.*, 2010 ▸) was reproduced using the ID32 sample preparation facility. As a first step, a clean Cu(111) surface was achieved by repeated cycles of sputtering and annealing the (111) surface of a Cu single crystal. The cleanliness of the surface was checked by AES, LEED and STM (Figs. 5*a*, 5*b* ▸). In a second step, Co was evaporated onto the clean surface at room temperature. In order to calibrate the Co evaporator, the QCM mounted on the manipulator was used. We then evaporated ∼20% of a monolayer onto the Cu(111) surface and subsequently characterized the surface by STM. The results are shown in Fig. 5(*c*) ▸. The vast majority of the deposited Co atoms cluster along the step edges. Only a small fraction are found on the terraces, probably at places where impurities on the surface served as nucleation centers. Starting from the oxidized Cu surface and with an uncalibrated evaporation source it took about 24 h to grow and characterize a sample ready to be studied with the X-ray beam.

**Figure 6:**
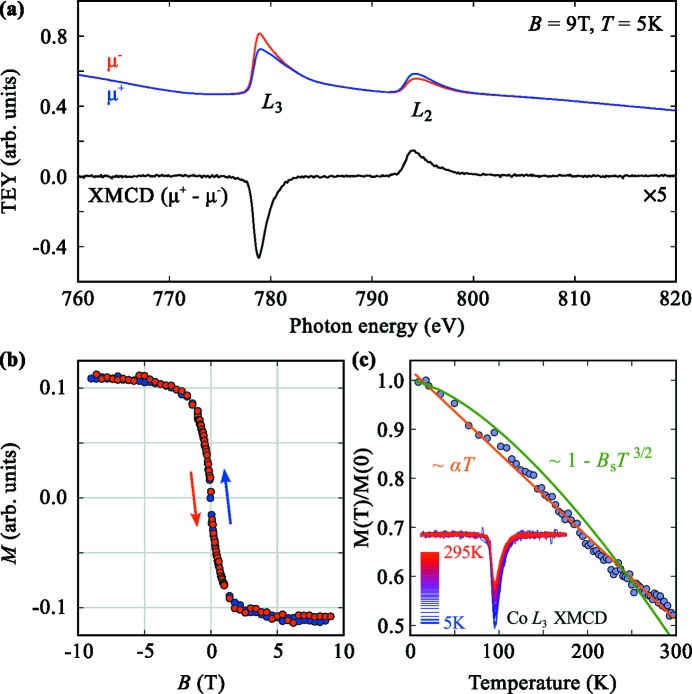
Magnetic characterization of the sample shown in Fig. 5 ▸. (*a*) Co *L*_2,3_ XMCD spectrum at 5 K and 9 T. (*b*) Co magnetization as a function of field at 5 K. (*c*) Co magnetization as a function of temperature at 9 T.

The Co/Cu(111) system has been magnetically characterized by measuring the field- and temperature-dependent XMCD signal at the Co *L*_2,3_-edges in TEY mode. The results are shown in Fig. 6 ▸. Panel (*a*) shows the XMCD signal at low temperature and high field. The XMCD signal was measured by applying the maximum field of 9 T along the beam and measuring the difference in the absorption for circular left and right polarized light. Two on-the-fly scans over an 80 eV energy window with 100 meV step size were taken for each polarization. In total the measurement took 5 min 42 s of which 5 min 20 s were data acquisition and 22 s were used for moving back motors and for changing the polarization. Given the low coverage of much less than one monolayer of cobalt on the surface this is a good example of the low-noise high-quality data that can be acquired with the setup in a few minutes. We obtain a signal-to-noise ratio of about 1.5 × 10^3^ in the pre- and post-edge region of the XAS spectra using the analysis method described in §3.3.

The Co magnetization curve at 5 K is shown in Fig. 6(*b*) ▸. It has been obtained by measuring at every field point the ratio between the absorption in the peak of the XMCD (778.8 eV) and before the edge (776 eV) for both circular polarizations. The magnetic field has been changed step by step. Measuring the ratio between peak and pre-edge absorption is necessary because the intensity of the TEY signal itself strongly depends on the magnetic field. A magnetization curve measured in this step-by-step fashion is typically obtained in one to a few hours depending on the field range and the desired number of points. The shown magnetization loop took 2 h 32 min. On-the-fly measurements of the magnetic signal while the magnet field is swept continuously are also possible and can be a significantly faster way of measuring magnetization loops (see §3.2).

The temperature dependence of the XMCD at 9 T is shown in Fig. 6(*c*) ▸. It has been obtained by slowly ramping the temperature from 5 K to 300 K at a rate of 1.25 K min^−1^ and continuously measuring the XMCD signal at the Co *L*_3_-edge (inset). The data clearly show that the Co magnetization fits a *T* ^1^ temperature dependence much better than a *T* ^3/2^ dependence. Such a linear *T* dependence has been interpreted in the past as a sign of weak or absent magnet interaction between the Co atoms and the surface (Binder & Hohenberg, 1974 ▸; Qiu *et al.*, 1992 ▸). In general, temperature-dependent element-specific measurements of the magnetization are a very important means to fully understand magnetic systems. The ID32 endstation is able to work at any fixed temperature or any temperature ramp between 5 K and 300 K with no effect on the noise level of the measurements. Efforts are being made to reduce the base temperature down to 3 K by improving thermal shielding of the sample. Several of the first user experiments heavily relied on temperature-dependent measurements over a wide *T* range (see, for instance, Baker *et al.*, 2015 ▸).

### 3.2. On-the-fly measurement of magnetization curves   

Element-specific magnetization curves *M*(*B*) are a standard request for XMCD setups. To obtain such curves the XMCD signal is measured as a function of the applied field at the absorption edge of interest. In order to obtain good quality curves with all experimental asymmetries removed, one typically measures the *M*(*B*) curves for the two circular polarizations, circular left (CL) and circular right (CR), and combines them to *M*(*B*) = *I*_XMCD_(+*B*, CL) − *I*_XMCD_(−*B*, CR), keeping in mind that (+*B*, CL) and (−*B*, CR) correspond to the same experimental helicity. For TEY measurements one has to additionally measure the signal just before the edge where there is no XMCD because the TEY signal itself can be strongly affected by the Lorentz forces in the magnetic field. We have implemented two different schemes to measure *M*(*B*) curves.

In the point-by-point scheme the magnetic field is changed in steps and the XMCD and the pre-edge signal are measured for both polarizations at every field point. The data shown in Fig. 6(*b*) ▸ have been obtained in that way. Point-by-point measurements usually give excellent data quality but they are slow due to the long time spent at each field point for changing polarizations and energy and for asking the magnet for the next field value. In the on-the-fly scheme the magnetic field is swept continuously and the signal is recorded on-the-fly. This scan is repeated up to four times to measure both the XMCD and the pre-edge signal for both circular polarizations. Because of the high rates at which the magnet can be swept, this measurement can be performed significantly faster than in the point-by-point scheme. In TFY the pre-edge signal does not need to be measured which reduces the total execution time by another factor of two. On the downside, the fact that the pre-edge and the XMCD signal are measured in two separate loops can sometimes be problematic for measuring TEY-detected *M*(*B*) curves when the dichroic signal is small or the field range is within ±100 mT where remnant fields of the magnet become important.

**Figure 7:**
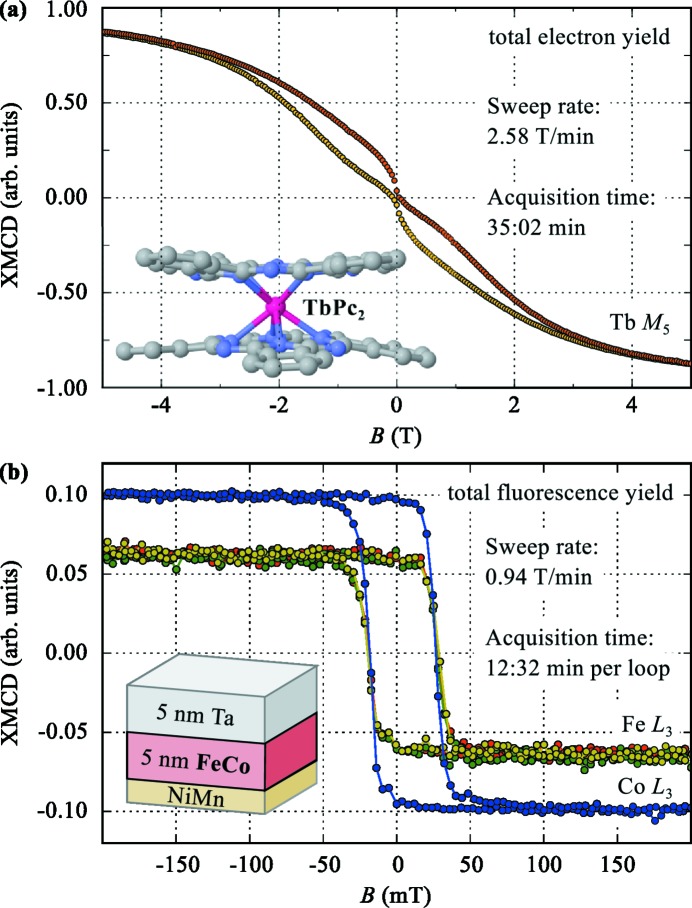
Element-specific magnetization loops measured on-the-fly for (*a*) a TbPc_2_ layer (M. Mannini, L. Poggini, M. Serri, R. Sessoli and P. Sainctavit, 23 September 2015) and (*b*) a buried FeCo layer.

In Fig. 7 ▸ we show two magnetization curves measured in the on-the-fly scheme. The sweep rate used in the on-the-fly scans is calculated from the requested number of data points and the integration time per point and is then automatically set to the magnet power supply. The data in panel (*a*) were taken by M. Mannini, L. Poggini, M. Serri, R. Sessoli and P. Sainctavit, 23 September 2015, at the Tb *M*_5_-edge from a thick layer of TbPc_2_ molecules. The sample has been prepared *ex situ* at LAMM-UNIFI by sublimation of a TbPc_2_ film of 100 nm on polycrystalline Au at 400°C and 5 × 10^−7^ mbar. The thickness has been monitored using a QCM and was verified by atomic force microscopy post-characterization. The measurements were performed by collecting the TEY signal at the Tb *M*_5_-edge as well as before the edge using both left and right circular polarization. The sample temperature was 7 K and the sample was rotated by 45° with respect to the light propagation direction. The external magnetic field was applied parallel to the light propagation direction. The obtained curve agrees well with curves previously reported for thick, amorphous TbPc_2_ layers (Malavolti *et al.*, 2013 ▸). The total time needed to obtain the *M*(*B*) curve was 35 min compared with 3.5 to 4 h to record a similar curve point-by-point. Fig. 7(*b*) ▸ shows *M*(*B*) curves from a ferromagnetic FeCo layer capped with 5 nm of Ta to prevent oxidation. The data were taken both at the Fe and the Co *L*_3_-edges recording the TFY signal on-the-fly. Each loop took 12 min 32 s compared with 52 min for a comparable point-by-point measurement, corresponding to a more than four times increase in data acquisition rate. The three consecutive loops taken at the Fe *L*_3_-edge overlap well, confirming the reproducibility of the on-the-fly measurements.

### 3.3. X-ray magnetic linear dichroism   

The possibility of applying a magnetic field perpendicular to the incident X-ray beam of up to 4 T allows swapping between XMCD and XMLD measurements in seconds. This is a big improvement compared with the previous system where we had to break vacuum and disconnect the sample preparation chamber in order to turn the entire magnet by 90°. XMLD is typically used to characterize the aligned moments in antiferromagnetic (AFM) systems where the net magnetic moment vanishes (van der Laan *et al.*, 1986 ▸). It is measured as the difference between the absorption of light with the **E** vector of the X-rays parallel and perpendicular to the direction of the magnetic field and the field being applied to align the magnetic domains. From an instrumental point of view it is very similar to XMCD measurements and all the possibilities of on-the-fly energy and magnetization scans can be used for XMLD measurements too.

**Figure 8:**
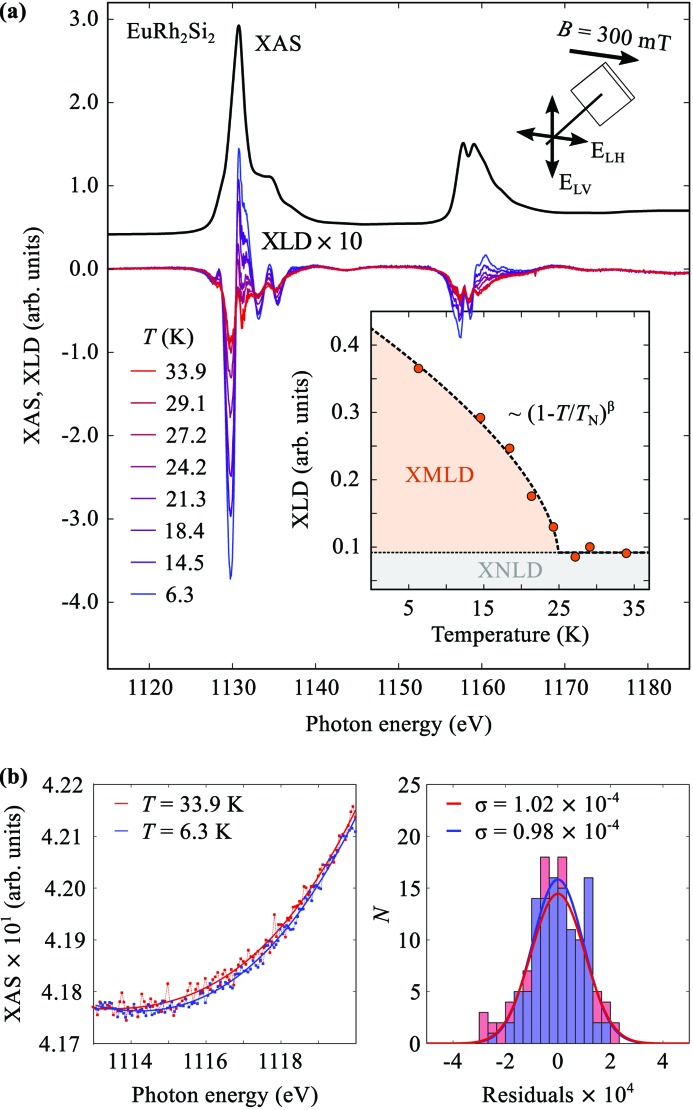
(*a*) X-ray linear dichroism at the Eu *M*_4,5_-edges of EuRh_2_Si_2_ as a function of temperature. (*b*) XAS data in the pre-edge region together with a smoothing spline through the data for *T* = 6.3 K (heater off) and *T* = 33.9 K (heater on). The right-hand panel shows a histogram of the distribution of the residuals together with the the best fit to a Gaussian for each temperature.

In Fig. 8(*a*) ▸ we show as an example an XMLD measurement for the well characterized antiferromagnet EuRh_2_Si_2_ (Seiro & Geibel, 2014 ▸). A small field of 300 mT was applied perpendicular to the beam direction in order to align the AFM domains, and the XMLD signal has been recorded as a function of temperature from low temperature to above *T*_N_ = 24.5 K. The angle between the *c*-axis and the incident beam was set to 70°. The inset shows the temperature dependence of the dichroic signal. The XMLD can be clearly separated from the temperature-independent X-ray natural linear dichroism (XNLD) caused by the orbital anisotropy. The data agree well with the known *T*_N_ of 24.5 K.

In order to obtain an estimate of the signal-to-noise ratio we analyzed the structureless pre-edge region of the XAS spectra as shown in Fig. 8(*b*) ▸. A smoothing spline, 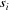, was fit to the experimental data points, 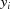, and the residuals, 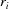 = 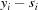, were plotted in a histogram. Fitting a Gaussian function to the distribution of the residuals, 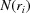, allowed us to obtain an approximation of the variance, 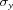, of the experimental data points. In Fig. 8(*b*) ▸ we show the respective analysis for the XAS spectra obtained at *T* = 6.3 K, with the heater switched off, and *T* = 33.9 K, with the heater on. The results show that the signal-to-noise ratio of the XAS data, which we define here as S/N = 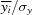, is independent of the VTI operation and was in this experiment approximately S/N = 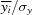 ≃ 4.2 × 10^−1^/1.0 × 10^−4^ ≃ 4 × 10^3^ in the pre-edge region. This S/N ratio is sufficient to detect XMLD signals of a few tenths of a percent of the absorption signal; 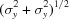 ≃ 6 × 10^−3^.

While XMCD signals are usually large at the transition metal *L*_2,3_- and rare-earth *M*_4,5_-edges, XMLD signals can be very small and overlap with a large XNLD signal in systems with notable orbital anisotropy. The very high signal-to-noise ratio and the possibility to easily change the direction of the magnetic field provide ideal conditions for XMLD or combined XMLD/XMCD studies of antiferromagnets.

## 4. Conclusions and outlook   

A new experimental endstation for X-ray magnetic dichroism experiments has been installed and commissioned at the ESRF soft X-ray beamline ID32. The system consists of a 9 T/4 T high-field magnet and a sample preparation system both connected in UHV. X-ray absorption experiments benefit from the very high signal-to-noise ratio at all temperatures and fields and from the fast data acquisition rate. Further improvements are foreseen for the near future. Preliminary tests show that the base sample temperature can be reduced down to 3 K by improved thermal shielding of the VTI. We also intend to install a Vortex-type fluorescence detector in addition to the photodiode to allow measurements in partial fluorescence yield. The setup is now in routine user operation, typically taking about 50% of the total beam time at the ID32 beamline of the ESRF.
